# Medio-Frontal and Anterior Temporal abnormalities in children with attention deficit hyperactivity disorder (ADHD) during an acoustic antisaccade task as revealed by electro-cortical source reconstruction

**DOI:** 10.1186/1471-244X-11-7

**Published:** 2011-01-12

**Authors:** Johanna Goepel, Johanna Kissler, Brigitte Rockstroh, Isabella Paul-Jordanov

**Affiliations:** 1 Department of Psychology, University of Konstanz, Konstanz, Germany

## Abstract

**Background:**

Attention Deficit Hyperactivity Disorder (ADHD) is one of the most prevalent disorders in children and adolescence. Impulsivity is one of three core symptoms and likely associated with inhibition difficulties. To date the neural correlate of the antisaccade task, a test of response inhibition, has not been studied in children with (or without) ADHD.

**Methods:**

Antisaccade responses to visual and acoustic cues were examined in nine unmedicated boys with ADHD (mean age 122.44 ± 20.81 months) and 14 healthy control children (mean age 115.64 ± 22.87 months, three girls) while an electroencephalogram (EEG) was recorded. Brain activity before saccade onset was reconstructed using a 23-source-montage.

**Results:**

When cues were acoustic, children with ADHD had a higher source activity than control children in Medio-Frontal Cortex (MFC) between -230 and -120 ms and in the left-hemispheric Temporal Anterior Cortex (TAC) between -112 and 0 ms before saccade onset, despite both groups performing similarly behaviourally (antisaccades errors and saccade latency). When visual cues were used EEG-activity preceding antisaccades did not differ between groups.

**Conclusion:**

Children with ADHD exhibit altered functioning of the TAC and MFC during an antisaccade task elicited by acoustic cues. Children with ADHD need more source activation to reach the same behavioural level as control children.

## Background

Children with ADHD have difficulties with cognitive control, working memory and response inhibition [1]. Response inhibition consists of two processes: (i) the capacity to suppress a prepotent response before or after its initiation, and (ii) the goal-directed behaviour from the interference of competing processes [2]. Antisaccades are one way to examine inhibition, as antisaccade tasks require the suppression of the automatic response to look towards a peripheral cue and to generate a saccade in the opposition direction instead [3]. Error rates during antisaccade tasks reflect the ability to inhibit a response, while saccadic reaction times (SRT) during correct trials reflect the duration of the underlying cognitive and motor processes. There is a growing body of literature on eye movement experiments comparing children with ADHD with control subjects [4]. Despite some inconsistencies, the general finding is that subjects with ADHD have an elevated number of direction errors during antisaccade tasks [5-13]. However, until now, no study has examined brain function during antisaccade tasks in ADHD, although this might lead to important new insight into the cortical mechanisms of behavioural inhibition and its dysfunction in ADHD.

Inhibition difficulties are not only relevant in the visual domain, where they have mostly been studied. Humans also redirect their gaze to locate the origin of a suddenly appearing noise, a tendency, which is already present in babies [14]. Still, until now, there is no study, which investigates pro- or antisaccades elicited by acoustic cues in children. Accordingly, it is unclear, which neuronal network underlies antisaccades following acoustic cues. There is a particular interest in analysing inhibition deficits following auditory cues in children with ADHD as a high number of children with ADHD have difficulties with acoustic tasks [15-17].

Electrophysiological and functional brain imaging studies have given insight into which cerebral areas are active during visual saccadic tasks. The Frontal Eye Fields (FEF), the Supplementary Eye Fields (SEF) and the Parietal Eye Fields (PEF) in the Posterior Parietal Cortex (PPC) are active when saccades are initiated. The Dorsolateral Prefrontal Cortex (DLPFC) and the Anterior Cingulate Cortex (ACC) with the Cingulate Eye Field are associated with "higher level", volitional and cognitive aspects of saccade control, specifically during antisaccades [18-26]. DLPFC shows activity during antisaccades that is not present during prosaccades [27]. Its activity seems to provide an inhibitory signal that precedes correct antisaccade performance [28-30]. Directional errors are therefore generally linked to frontal dysfunctions. The ACC is involved in the executive control of attention and plays an important role in visual antisaccade performance [24,31-33]. Given that children with ADHD have difficulties with response inhibition and make more antisaccade errors than children without ADHD, one might assume that activity of frontal structures involved in the generation of antisaccades is altered. Disturbed functioning of Prefrontal Cortex, ACC, and striatum are also thought to underlie other executive function deficits in ADHD [34]. This is in line with the aetiological theory that ADHD results from structural and functional changes in a fronto-subcortical network [34-36].

The first aim of the present study was to investigate how children with and without ADHD differ in brain activation during an antisaccade task. The second aim was to investigate, whether children with ADHD have comparable inhibition difficulties when cues are visual and acoustic.

## Methods

### Participants

Sixteen children with ADHD and sixteen children without ADHD were investigated. Children with ADHD were recruited at two child psychiatric outpatient clinics, diagnoses being made by the head psychiatrist and his/her team of psychologists based on questionnaires, anamnestic biographical interviews and psychometric tests. Control children were recruited at a local school. However, data of seven children with ADHD and data of two control children had to be discarded due to insufficient data quality (too many movement artefacts). Data of nine children with ADHD (mean age 122.44 ± 20.81 months, boys only) and 14 healthy control children (mean age 115.64 ± 22.87 months, three girls) were further analysed. All but one child with ADHD were diagnosed with ADHD combined type; the remaining child was diagnosed with ADHD primarily inattentive type. All children were investigated off medication. Three children with ADHD who were prescribed with methylphenidate refrained from taking it at least 24 hours before the experiment in concordance with their respective psychiatrist and their parents. All children with ADHD had at least one comorbid disorder (mostly specific developmental disorder of motor function) and 44% had at least two comorbid disorders (mostly specific developmental disorders of scholastic skills). Control children did not have any clinically relevant diagnoses or took any medication as reported by the parents.

### Procedure

Children and parents were shown the laboratory equipment and the task was explained to them. They then signed informed consent forms (according to the Helsinki declaration [37]). Parents were asked to fill in an ADHD symptom checklist [38], an auditory processing disorder (APD) checklist [39] and a routine questionnaire while children completed the Edinburgh-Handedness-Inventory [40]. To ensure within-normal hearing levels, children's hearing thresholds were determined for frequencies 500, 1000, 2000 and 4000 Hz in an acoustically shielded room. Children were then shown a computerised, animated explanation of the task, which included examples and four training trials. To ensure that all children were motivated and perceived themselves as successful, children were told that they would be able to collect four "cartoon dogs" on the computer screen if they performed well (the dogs always appeared after fixed intervals) which would then allow the children to pick a small gift from a "treasure chest" after the experiment. Children were additionally compensated with 20 Euros at the end of the experimental session.

For the EEG experiment, children were comfortably seated in a chair, their heads resting on a chin rest 500 mm away from the computer monitor. Headphones were put on and the 30 min - experiment was started after impedance measurement. After the EEG experiment intelligence was assessed by the Coloured Progressive Matrices (CPM) [41].

### Task

Participants were instructed to generate saccades in response to visual or acoustic cues. The nature of the required saccade depended on the instruction. Saccades could either be directed towards the cue (prosaccade) or away from the cue (antisaccade). Visual cues, consisting of yellow dots that filled one of four empty circles, could appear "near" (6°) or "far" (12°) and left or right of the fixation cross for 1000 ms. Acoustic cues were 1000 Hz sine tones presented for 1000 ms that were perceived either "far" left/right (90°) or "near" left/right (45°, see the description below). Children were explained that in response to "near" acoustic cues they should generate saccades towards the 6° circle, and upon "far" to make saccades towards the 12° circle. Cues could either appear 200 ms after extinction of the fixation cross (gap) or with a 200 ms overlap with the fixation cross. Random combinations of the following within-group factors were presented throughout the experiment: cue modality (visual vs. acoustic), direction (right vs. left), type (anti- vs. prosaccade), distance (near (6° visual, 45° acoustic) vs. far (12° visual, 90° acoustic)) and delay (gap vs. overlap). Nine runs of each combination resulted in a total of 288 trials. This random design was chosen to avoid ceiling effects and enable better group differentiation.

After trial 96, 129, 259 and 288 children were shown a motivation picture with 1, 2, 3 and 4 dogs, respectively. A pause-signal appeared after 144 trials indicating that children could take a short break. The length of the break was determined by the children.

**Figure 1:**
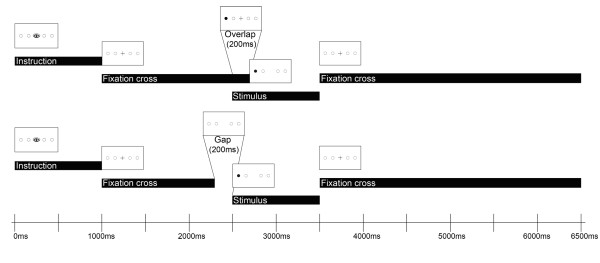
**Temporal structure of an exemplary trial (visual prosaccade)**. Top: Overlap-condition, bottom: Gap-condition. Every trial started with the presentation of an instruction slide for 1000 ms (prosaccades: picture of an eye or ear; antisaccades: picture of a crossed-out eye or ear) followed by a fixation cross. Stimulus onset was at 2500 ms in both conditions. In the gap condition, the fixation cross disappeared 200 ms before stimulus onset, while in the overlap condition the fixation cross disappeared 200 ms after stimulus onset. After stimulus offset at 3500 ms the fixation cross was presented again for 3000 ms.

Each trial began with a 1000 ms instruction slide depicting the nature of the required saccade by a prominent symbol the meaning of which had been explained to the children beforehand (see procedure above). Each trial lasted 6500 ms (see Figure 1 for a schematic overview).

### Equipment and Recordings

Cues were presented with the software Presentation (Neurobehavioral Systems, Inc.). Visual cues were generated within Presentation. Sine tones were generated with Adobe Audition 2.0^®^. The effect of sound lateralisation was created by intensity and phase differences between the left and right channel. The impression of a 90° lateralisation to either direction was created by attenuating the contra-lateral channel by 3.62 dB and shifting its onset by 6.5 μs. The impression of a 45° lateralisation was created by attenuating the contralateral channel by 2.8 dB and delaying its onset by 1 μs.

Stimuli were presented with a PC Dell precision 390 with Intel ^® ^Core ™ 2CPU 2.13 Hz-processor with 2 GB Ram operating system on a monitor with 365 × 270 mm resolution (Samtron 96 BDF) and via stereo headphones (Sennheiser HD 280 pro (64Ω)).

Electrical brain activity was measured using EEG. Recording was done with a 257 channel system from EGI Electrical Geodesics Inc. using NetStaion^TM12 ^on a Mac OSX with 1,25 GHz PowerPC G4 processor and 1 GB DDR SD RQM. Sample rate was 250 Hz and an online filter of 100 Hz lowpass and 0.1 Hz highpass were applied.

### Data analysis

**Figure 2:**
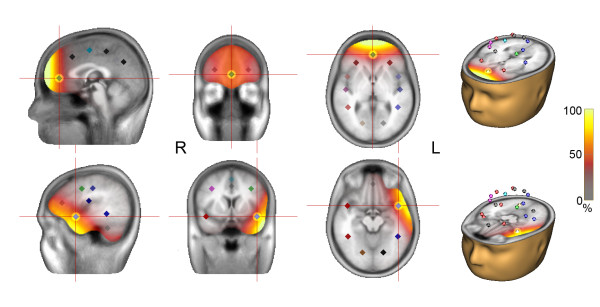
**Sensitivity map of the MFC (top) and the TAC left (bottom)**. Location and sensitivity of the MFC and TAC source in sagittal, transversal and horizontal view

Data were analysed with BESA software (Brain Electrical Analysis, version 5.2.4.52, MEGIS Software GmbH, Graefelfing, Germany). Vertical and horizontal eye movements artefacts (blinks and saccades) were systematically removed using an algorithm implemented in BESA [42,43]. For each condition, data were segmented into epochs from 500 ms pre to 2000 ms post stimulus (notch filter at 50 Hz). For the identification of saccades, data were filtered digitally from 0.01-8 Hz (6 dB/octave forward and 12 dB/octave zerophase). The percentage of correct saccades was determined and saccade latency was measured to the nearest sampling point. Saccades with latencies <80 ms were excluded, as they can be classified as anticipations rather than responses [44]. Next, unfiltered response-locked averages of antisaccades (merged across direction, distance and delay to gain higher statistical power and more averages for source reconstruction) were generated i.e. epochs (500 ms pre and 500 ms post response) were exported, which were centred at saccade onset. Source analysis was carried out with a 23-source-model (generated on the basis of talairach coordinates of structures known to be involved in saccade generation), data being filtered digitally from 0.1-30 Hz (6 dB/octave forward and 24 dB/octave zerophase). The source montage was generated to cover activity of structures relevant for the processing and production of saccades (FEF, DLPFC, PPC - left and right, SEF, Frontal Midline (FM) and Medio-Frontal Cortex (MFC)). Further, sources were placed that covered activity of structures relevant for the processing of acoustic and visual stimuli (Supplemental Temporal Cortex (STC), Temporal Parietal Cortex (TPC), Temporal Anterior Cortex (TAC) and Occipital Cortex (OCC) - left and right). Additional sources of no interest (Cerebellum (CB) - left and right) were placed to increase the sensitivity of the sources of interest. The sensitivity of a source describes its ability to pick up the activity generated by the brain volume of interest. Source sensitivity is dependent on the position of the source in the brain model, the number of sources in the montage, as well as the distance between the sources. The sensitivity of relevant sources was carefully tested with sensitivity maps in BESA (see Figure 2 for the sensitivity map). The output of a source montage is each individual source's activity over time. Source positions in space are fixed.

### Statistical analysis

Only antisaccades were analysed, as the leading question of the present article concerned response inhibition. Saccadic reaction times (SRTs) and the percentage of correctly generated antisaccades (merged across direction, distance and delay) were compared between groups using Statistica (StatSoft, Inc., 2003). T-tests or Mann-Whitney-U tests were computed after testing for normal distribution of the dependent variables using Shapiro-Wilks-W-test. Scores of questionnaire data were analysed accordingly. In order to objectively identify time-windows, throughout which the experimental groups differed in activity of one or more sources, non-parametric cluster-based analysis of EEG source data was performed using FieldTrip, an open-source signal processing toolbox for Matlab (Donders Institute for Brain, Cognition and Behaviour, Radboud University Nijmegen, The Netherlands. http://www.ru.nl/neuroimaging/fieldtrip). Groups were compared for each sampling point and each source via independent t-tests. In order to prevent chance-findings, data were re-shuffled 1000 times using a cluster-based Monte-Carlo randomization.

This method effectively controls for multiple comparisons [45]. Clusters (here: clusters of sampling points) were defined as significant when the probability of observing larger effects in the shuffled data was below 5%. As response inhibition takes place before the onset of the saccade and in accord with already existing findings [29,30], data analysis was carried out for the time-windows -230 ms until -120 ms before response and -120 ms until 20 ms after response.

## Results

### Sample characteristics

Groups did not differ in age (*t*(21) = 0.689, *p *= .499) or gender distribution (*χ*^*2*^(1) = 2.22, *p *= .135). Children with and without ADHD had comparable intelligence scores as measured by the CPM (ADHD: 71.00 ± 29.97 percentile rank, Control: 66.15 ± 29.84 percentile rank; *t*(19) = 0.361, *p *= .722). Children with and without ADHD had hearing sensitivities of 20 dB or better in each ear for all measured frequencies [46]. Groups did not differ from each other (see table 1).

**Table 1:** Results hearing levels

			ADHD (n = 9)		Control (n = 14)				
							
Side tested	Test	Frequency (Hz)	Mean	SD	Mean	SD	t/Z- value	df	p
	t-test	500	4.67	5.05	3.50	4.15	0.605	21	0.552
Right	t-test	1000	1.56	4.98	0.21	3.93	0.721	21	0.479
	t-test	2000	-0.89	4.83	-0.79	4.92	-0.049	21	0.961
	t-test	4000	0.33	5.36	-0.93	6.81	0.469	21	0.644

	t-test	500	3.00	7.45	3.36	5.42	-0.133	21	0.895
Left	MWU	1000	-1.33	8.02	-0.86	6.77	-0.031	21	0.975
	MWU	2000	-2.67	5.55	0.07	8.40	-0.661	21	0.508
	MWU	4000	-2.00	6.08	-0.43	9.49	-0.504	21	0.614

Children with ADHD had higher values than control children for both subscales of the ADHD questionnaire (see table 2). Groups also differed on the subscales Speech Perception and Auditory Memory of the APD questionnaire (see table 2).

**Table 2:** Results parental ratings of ADHD/APD symptoms

				ADHD			Control					
								
Symptoms	Sub-scales	Test	n	Mean	SD	n	Mean	SD	t/Z-value	df	p	
ADHD	Inattention	MWU	9	34.00	7.38	14	14.71	2.40	3.874	21	0.000	***
	Hyperactivity/Impulsivity	MWU	9	3.09	0.67	14	1.34	0.22	3.969	21	0.000	***

	Speech Perception	t-test	9	1.89	0.73	13	1.29	0.25	2.767	20	0.012	*
	Auditory Discrimination	MWU	9	1.38	0.72	14	1.14	0.23	0.787	21	0.380	
APD	Sound Localisation	MWU	9	1.27	0.53	14	1.01	0.05	1.134	21	0.086	
	Hearing in background noise	MWU	9	1.63	0.78	14	1.48	0.41	0.157	21	0.874	
	Auditory Memory	MWU	9	1.81	0.65	14	1.30	0.42	2.331	21	0.019	*
	Auditory Hypersensitivity	t-test	9	2.77	0.64	13	2.48	0.62	1.058	20	0.303	

### Saccadic reaction and latencies

Groups did not differ regarding correct antisaccade reactions in the visual condition (ADHD 50.52 ± 16.54% correct, Control 48.84 ± 20.53% correct, *t*(21) = 0.205, *p *= .839) and in the acoustic condition (ADHD: 57.20 ± 12.88% correct, Control: 65.38 ± 12.32% correct, *t*(21) = -1.527, *p *= .142).

There were neither group differences in antisaccade latency in the visual condition (ADHD: 493.36 ± 196.43 ms, Control: 441.00 ± 146.65 ms, *Z*(21) = 0.504, *p *= .614), nor in the acoustic condition (Antisaccades: ADHD: 696.25 ± 258.34 ms, Control: 639.94 ± 226.71 ms, *t*(21) = 0.551, *p *= .588).

### Pre-saccadic brain activity

**Figure 3:**
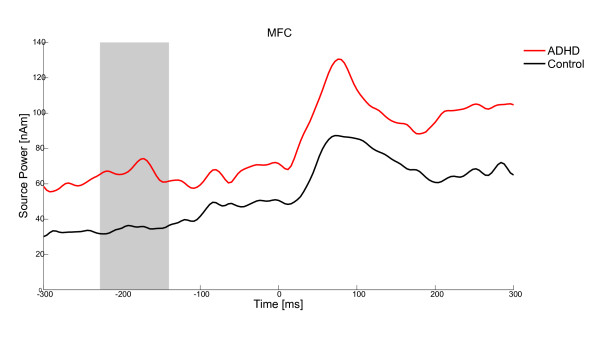
**Group effect for the dependent variable source power of correct antisaccades in the MFC**. Source activity 300 ms before saccade onset until 300 ms after saccade onset in children with ADHD (red) and control children (black) in the MFC; The grey bar highlights the time of significant group difference.

**Figure 4:**
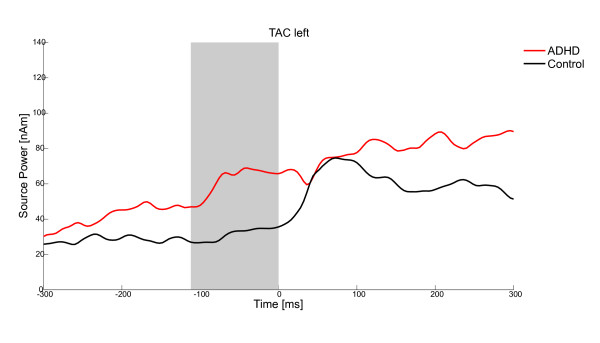
**Group effect for the dependent variable source power of correct antisaccades in the TAC left**. Source activity 300 ms before saccade onset until 300 ms after saccade onset in children with ADHD (red) and control children (black) in the TAC; The grey bar highlights the time of significant group difference.

A significant group difference was identified for the acoustic antisaccade condition between 228 and 140 ms before antisaccade onset (*t*(21) = 74.707, *p *< .05) in the MFC source and at 112-0 ms before antisaccade onset (*t*(21) = 76.294, *p *< .05) in the TAC left source. Children with ADHD showed higher source activity than control children (MFC: ADHD: 67.09 ± 40.16 nAm, Control. 34.59 ± 13.49 nAm, see Figure 3; TAC left: ADHD: 61.83 ± 31.80 nAm, Control 31.34 ± 20.18 nAm, see Figure 4).

In contrast, no significant group differences were revealed in the visual antisaccade condition in either of these sources or any other source.

## Discussion

Aim of this study was to investigate differences in response inhibition and corresponding brain activity between children with and without ADHD. Response inhibition was measured in an antisaccade task where saccades were either elicited by acoustic or visual cues.

The main finding of the study was that children with and without ADHD differed in brain activity when saccades were elicited by acoustic cues. Children with ADHD had a higher source activity than control children in the MFC source between -228 and -140 ms and in the left-hemispheric TAC source between -112 and 0 ms before saccade onset. These time windows overlap with the critical period for response inhibition in visual antisaccade tasks [29,30,47].

### Behavioural data

No group differences regarding the correctness of saccade execution were found in the present study. Other studies on antisaccades using only visual cues revealed an elevated number of direction errors in children with ADHD [4], indicating that these children are less able than control children to inhibit inappropriate responses. However, there are also studies in line with the present findings [48-50] without group differences. The random design of experimental presentation in the present study was chosen to increase task difficulty in order to differentiate between the groups. However, it might have been the case that the task was equally more difficult for both, control children and children with ADHD, as supplementary task switching between pro- and antisaccades is required [12,51], thus concealing group effects.

Another explanation for the negative finding of behavioural group differences might be related to the age range of the children in the present study. Rothlind and colleagues [50] investigated a group of children with a similar age range. The mean age of their ADHD group was 10.5 ± 2.4 years (range: 6.9 - 13.9 years), mean age of the control group was 9.9 ± 2.8 years (range: 6.8 - 14.4 years). As in the present study, Rothlind and colleagues did not find any group differences in saccadic errors. Other studies have used groups of children with a smaller age-range and were able to find more errors in children with ADHD [5,6,8,10-12]. A reason might be that boys younger than 11 years have difficulty with oculomotor inhibition in general [52,53]. However, a study with younger children has also found differences between children with and without ADHD [10] and thus questions the assumption of a general oculomotor inhibition deficit in younger children. Finally the subtype of ADHD might be an influencing factor on performance in saccade tasks. Children with ADHD combined type made more antisaccade errors than control children, while no group differences were found between children with ADHD inattentive type and control children [12]. In the present study eight of nine children with ADHD had the diagnose ADHD combined type. Thus, ADHD subtype is not likely to have influenced the response pattern in the present study.

As for saccadic correctness, no group differences were found for SRTs in the present study. The latency of correct antisaccades was not investigated in all saccade studies and results are inconsistent. Some studies found slower antisaccade latencies in children with ADHD compared with control children [5-10]. Other studies found no group differences in antisaccades latencies [12,50], which is in line with the present result.

Thus, it is still unclear why no group differences were found in the rate of correct saccades and its latencies. The small sample size - which resulted from the fact that only ADHD children off medication were included - and the relatively big age range seem to be the most likely explanation. However, an absence of behavioural differences reduces ambiguities in the interpretation of any effects in brain measures.

### Pre-saccadic brain activity

Indeed, source activation differed between groups in the acoustic condition. Children with ADHD had higher activation of the MFC and the left-hemispheric TAC compared to control children during time-windows likely to reflect response inhibition. MFC includes parts of the dorsal ACC, which is connected with the prefrontal cortex and parietal cortex as well as the motor system and the frontal eye fields [54-56]. It is crucially involved in the executive control of attention. The ACC plays an important role in visual antisaccade performance [24,31-33] and ACC activity seems to be altered in patients with ADHD [57-60]. In the present study, children with ADHD had higher activity in the MFC source than control children preceding an auditory antisaccade. Still, behavioural performance, i.e. the percentage of correctly executed saccades did not differ between the groups. It thus appears that children with ADHD needed more activation of the MFC to reach the same level of response inhibition as control children. The present results were found only when saccades were elicited by acoustic cues. Still, a comparable pattern of brain activation results was found in studies investigating response inhibition in a visual go/nogo task design [35,61,62]. The present results are also in line with a meta - analysis [35], which concluded that there are two brain areas, in which ADHD patients have significantly more activation than controls: the medial frontal gyrus and the right secondary somatosensory area.

Activation of the left TAC source was higher in children with ADHD than in control children preceding antisaccades. Results from other experiments regarding temporal lobe activity during cognitive tasks are inconsistent. There seems to be some evidence of dysfunction and also of compensatory use of the temporal lobes in ADHD [63]. However, the current finding is in line with a go/nogo study in which children with ADHD showed more activation than the control children in the middle/inferior/superior temporal gyrus [64]. This might be also related to structural abnormalities in children with ADHD [36]. Castellanos and colleagues [65,66] showed that children with ADHD have a reduced volume of frontal and temporal gray matter, caudate, and cerebellum. These volume reductions were related with measures of symptom severity in an ADHD sample [65,67]. Another study detected reduced brain volumes in the lateral anterior and midtemporal cortices bilaterally [68]. Lateral temporal and parietal regions are part of the cross-modal association cortex, which also includes the DLPFC. This system integrates information from lower order sensory systems into higher order rules and functions. It is assumed that these regions together - beside their anatomical interconnection - form a broadly distributed action-attention system that supports the maintenance of attentional focus and successful inhibition [68-70]. It might be speculated that because of the smaller volume of the temporal cortex, children with ADHD showed more reflexive reaction to acoustic cues. Because of that, more frontal activation might have been needed as well in order to control behavioural output.

Finally, group differences in brain activation during acoustically elicited antisaccades are in line with auditory deficits (in Speech Perception and Auditory Memory) as detected in the APD questionnaire in the present study. The results are also in line with a suggested symptom overlap of children with ADHD and children with APD [71-74]. APD is characterised by disturbed hearing despite a normally functioning periphery. Typical symptoms are poor recognition, discrimination, separation, grouping, localisation, ordering of non-speech sounds and difficulties with acoustic tasks when competing acoustic signals are present [75,76]. Both, children with APD and children with ADHD, have difficulty paying attention and remembering information presented orally, are easily distracted, have difficulty following complex auditory directions or commands, and show low academic performance. The present results also demonstrate that acoustic processing should be a focus of interest in ADHD research. Knowing more about alterations of the auditory systems and according consequences might enable better differentiation of the ADHD/APD diagnosis.

In summary, both structures - MFC and the left-hemispheric TAC - are part of functional brain areas involved in attention and response inhibition, and seem to be functionally or structurally altered in children with ADHD.

Against expectations, no differences in brain activity were found in the visual antisaccade condition. There might be many contributing factors such as sample size, task design, and age range, as mentioned above. It is not possible to directly compare the present results to previous findings, as no other studies have investigated brain activation during antisaccades in children with ADHD. However, it should be noted that there are inconsistent findings in imaging studies of other visual inhibition tasks. Some studies reported that ADHD children exhibit a smaller P3 amplitude than control children [60,77-79], and showed lower activation of inferior prefrontal cortex and other brain regions [35,80,81]. Other authors found increased activation in prefrontal brain regions [61,62] and in the medial frontal gyrus respectively [35]. Again, it is difficult to compare studies using different inhibition tasks. More research with bigger sample sizes and a smaller age range are needed to answer to the question if there are differences in brain activity between children with and without ADHD during visually cued antisaccades.

## Conclusion

In sum, the present study for the first time provides insight in the cortical network underlying the production of antisaccades elicited by acoustic stimuli in children with and without ADHD. While no group differences were found when visual cues were used, results showed that functioning of the Anterior Temporal Lobe and Medio-Frontal Cortex is altered in children with ADHD when acoustic cues are used to trigger antisaccades. The present results support the hypothesis that cortical structures underlying response inhibition are more active in children with ADHD to achieve the same behavioural output as children without ADHD, possibly as a compensatory mechanism.

## Competing interests

The authors declare that they have no competing interests.

## Authors' contributions

JG carried out the subject selection, data acquisition, data processing, statistics and the preparation of the manuscript. Substantial contribution to study design, data analysis and the maniscript was made by JK. BR supervised the study and offered advice on data analysis and manuscript preparation. The study was designed by IPJ. Additionally she carried out statistics and corrected the manuscript.

All authors read and approved the final manuscript.

## Pre-publication history

The pre-publication history for this paper can be accessed here:

http://www.biomedcentral.com/1471-244X/11/7/prepub
